# Where and When To Inject Low Molecular Weight Heparin in Hemodiafiltration? A Cross Over Randomised Trial

**DOI:** 10.1371/journal.pone.0128634

**Published:** 2015-06-15

**Authors:** Annemieke Dhondt, Ruben Pauwels, Katrien Devreese, Sunny Eloot, Griet Glorieux, Raymond Vanholder

**Affiliations:** 1 Department of Nephrology, Ghent University Hospital, Ghent, Belgium; 2 Coagulation Laboratory, Department of Clinical Chemistry, Microbiology and Immunology, Ghent University Hospital, Ghent, Belgium; Medical University of Graz, AUSTRIA

## Abstract

**Background and Objective:**

Low molecular weight heparins (LMWHs) are small enough to pass large pore dialysis membranes. Removal of LMWH if injected before the start of the session is possible during high-flux dialysis and hemodiafiltration. The aim of this study was to determine the optimal mode (place and time) of tinzaparin administration during postdilution hemodiafiltration.

**Study Design, Setting, Patients:**

In 13 chronic hemodiafiltration patients, 3 approaches of injection were compared in a randomised cross over trial: i) before the start of the session at the inlet blood line filled with rinsing solution (IN_0_), ii) 5 min after the start at the inlet line filled with blood (IN_5_) and iii) before the start of the session at the outlet blood line (OUT_0_). Anti-Xa activity, thrombin generation, visual clotting score and reduction ratios of urea and beta2microglobulin were measured.

**Results:**

Anti-Xa activity was lower with IN_0_ compared with IN5 and OUT_0_, and also more thrombin generation was observed with IN_0_. No differences were observed in visual clotting scores and no clinically relevant differences were observed in solute reduction ratio. An anti-Xa of 0.3 IU/mL was discriminative for thrombin generation. Anti-Xa levels below 0.3 IU/mL at the end of the session were associated with worse clotting scores and lower reduction ratio of urea and beta2microglobulin.

**Conclusions:**

Injection of tinzaparin at the inlet line before the start of postdilution hemodiafiltration is associated with loss of anticoagulant activity and can therefore not be recommended. Additionally, we found that an anti-Xa above 0.3 IU/mL at the end of the session is associated with less clotting and higher dialysis adequacy.

**Trial Registration:**

Clinicaltrials.gov NCT00756145

## Introduction

For renal replacement strategies such as hemodialysis and hemodiafiltration, anticoagulants are required to prevent clotting of the extracorporeal circuit. Over-anticoagulation can be associated with hemorrhage and prolonged bleeding at the needle insertion sites. Insufficient inhibition of the coagulation cascade can lead to premature interruption of the dialysis session. More subtle under-anticoagulation could lead to clotting of some fibers with a decrease in membrane exchange surface area eventually resulting in a reduction in dialysis efficiency.

Low molecular weight heparins (LMWH) are often preferred to unfractionated heparin [1] because of the ease of a single injection at the start of the session; they are widely used anticoagulants for hemodialysis [2]. LMWHs have mean molecular weights (MW) between 3600 and 6500 D. Therefore, especially if unbound, they can pass high-flux dialysis membranes. So when LMWHs are administered at the inlet blood line before the lines are filled with blood, they may disappear in the dialysate compartment. Tinzaparin is a LMWH obtained by enzymatic depolymerization of unfractionated heparin. The mean MW of the chains is 6500 D.

The aim of this study was to determine the optimal mode of administration of LMWH as anticoagulant during postdilution hemodiafiltration.

Three approaches of administration of tinzaparin were compared: i) before the start of the session at the inlet blood line filled with rinsing solution (IN_0_), ii) 5 min after the start of the session at the inlet blood line filled with blood (IN_5_) and iii) before the start of the session at the outlet blood line (OUT_0_). Anti-Xa levels, thrombin generation, visual appreciation of clotting of the circuit and dialysis performance measured as RR of a small and middle MW molecule were considered.

## Materials and Methods

### Study design

**Fig 1:**
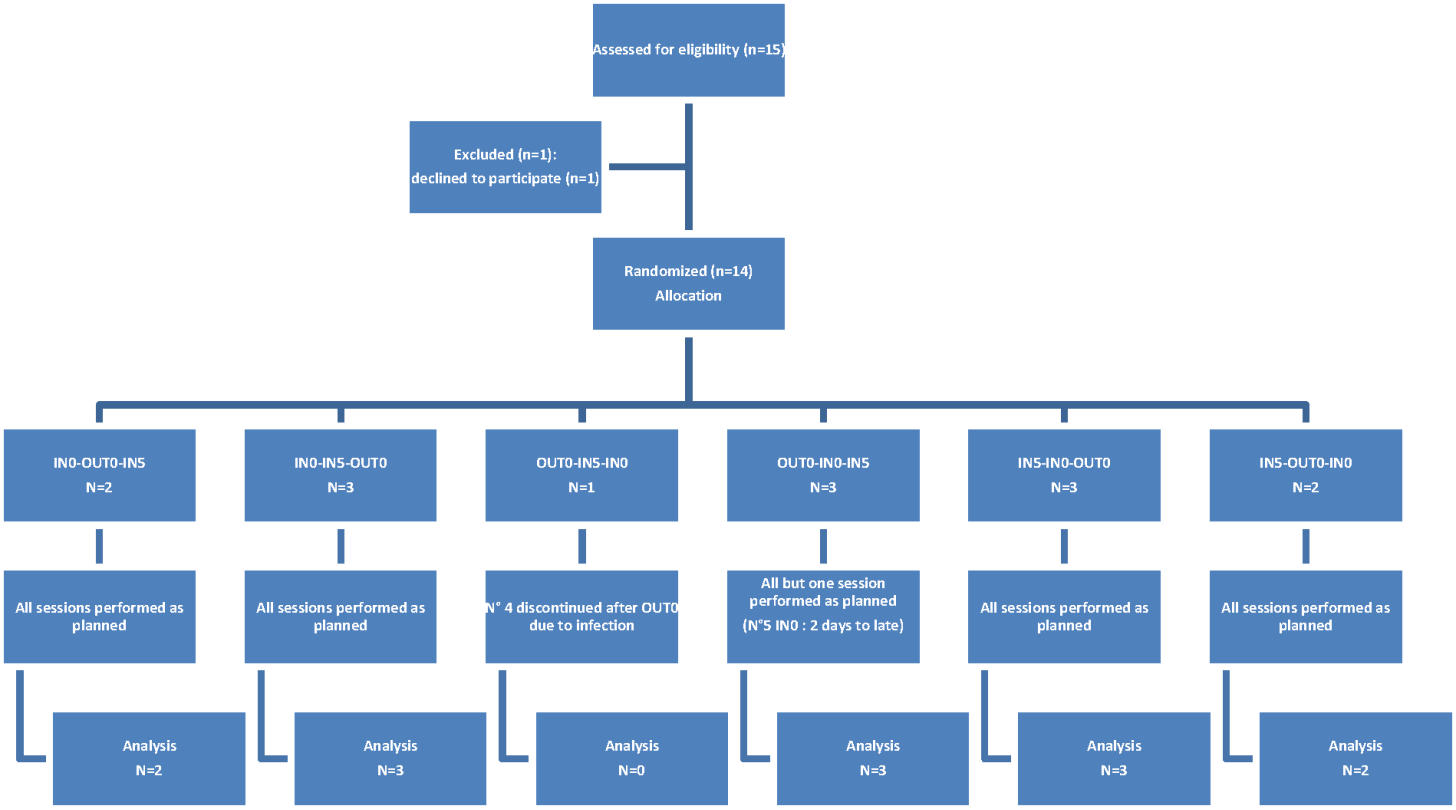
Study flow chart: generation of allocation sequence, enrollment and assignment was done by AD.

The protocol for this trial and supporting CONSORT checklist are available as supporting information; see S1–S3 Texts. The study flow chart of this cross over randomised trial is summarized in Fig 1. In each patient, three options were studied in cross over: i) administration of tinzaparin at the inlet blood line just before the start of the blood pump (IN_0_), ii) administration at inlet blood line 5 minutes after the detection of blood by the blood detector (IN_5_) and iii) administration at the outlet blood line just prior the start of the blood pump (OUT_0_). The dose of tinzaparin remained unchanged throughout the study. The sequence of the sessions per patient was assigned randomly. For each patient, the experimental sessions were performed with a 1-week interval at the same dialysis day of the week, either first, second, or third session of the week. Blood was sampled from the vascular access at the start of the session, before heparin injection and from the inlet dialyzer blood line at times 10, 30, 120, 180 and 240 min. Also transmembrane pressure (TMP), prefilter pressure, pressure in inlet and outlet bloodline were registered at 10, 30, 60, 90, 120, 150, 180, 210 and 240 min. Primary end-point was the anti-Xa activity at the end of the session. Secondary end-points were: anti-Xa activity at the start, after 30, 120 and 180 min, ETP at the start and after 30, 120, 180 and 240 min, dialysis efficiency measured as reduction ratio of urea and beta2microglobulin after 10, 180 and 240 min. Other secondary end-points were visual clotting scores at the end of the session and pressure measurements along the circuit.

### Patients

Fourteen patients treated with chronic hemodiafiltration at the Ghent University Hospital were included in the study from september 2008 until march 2010 (Fig 1). Inclusion criteria were chronic kidney disease stade V, older than 18 years and hematocrit above 30%. Exclusion criteria for the study were: active bleeding, infection or malignancy, hepatic failure, thrombocytopenia below 120,000/μL, treatment with vitamin K antagonists, administration of heparin or anticoagulants for other reasons than anticoagulation for dialysis, allergy towards heparin. Thirteen chronic hemodialysis patients (8 males, 5 females, median age 74 years (69–80 interquartile range)), completed the study. One patient was prematurely excluded due to an unrelated infectious adverse event. The median and interquartile range of body weight was 68 (63–81.5) kg. The renal diagnoses were diabetic nephropathy (n = 4), renal vascular disease (n = 3), chronic interstitial kidney disease (n = 2), and other (n = 4). As dialyis anticoagulant, tinzaparin (Innohep, Leo Pharmaceutical Corp, Ballerup, Denmark), with median and interquartile range of 4500 (3500–4500) IU was routinely used. Before the study, tinzaparin was injected in the afferent blood line shortly after the start of the session. The doses had been defined prior to the start of the study, based on the presence or absence of visible clotting of membrane and circuit and/or prolonged bleeding after dialysis.

Four patients received antiplatelet therapy, either aspirin (n = 3) and/or clopidogrel (n = 2). Residual renal function measured as average of creatinine and urea clearance was 3.0 (0–5.2) mL/min.

### Ethics statement

Approval of the local ethics committee (Ghent University Hospital ethics committee) was obtained, as well as written informed consent of all patients. The study was registered in clinicaltrials.gov as NCT00756145.

### Hemodiafiltration

The hemodiafiltration treatments were performed with AK200 ULTRA S (Gambro, Lund, Sweden) dialysis machines with BL200BD and BL208BD (Gambro, Medolla, Italy) as inlet and outlet blood lines. As dialyzer, single use Helixone membranes (FX800, Fresenius Medical Care, Bad Homburg, Germany) were used. No heparin was added in the rinsing solution of the circuit.

As anticoagulant, prefilled syringes containing tinzaparin were used in the same dose as before the study. The hemodiafiltration sessions lasted 240 minutes with the following characteristics: effective blood flow 300 mL/min, dialysate flow 600 mL/min, volume controlled postdilution hemodiafiltration with an effective convection rate of 75 mL/min.

During the sessions no other intravenous medication was administered.

At the end of the session, the circuit was rinsed by means of an online restitution program.

### Sample collection

Blood samples were collected in citrated tubes (Venosafe 3.6 ml, 0.109 M buffered sodium citrate, Terumo Europe, Leuven, Belgium) for measurement of anti-Xa, antithrombin and thrombin generation; in tubes with gel and clotting activator (VenosafeAutosep, Terumo) for CRP, urea and beta2microglobulin and in K-EDTA tubes (BD Plymouth, UK) for hematocrit.

Hematocrit and CRP were measured immediately; serum and plasma samples for all other determinations were kept at −80°C for further analysis.

### Analytical techniques

CRP was measured through a particle enhanced immunoturbidimetric assay (CRPLX, Roche Diagnostica, Mannheim, Germany).

Hematocrit was determined by centrifugation (Heraus Centrifuge, Labofuge 400).

Beta2microglobulin concentrations were quantified using an ELISA kit (Orgentec Diagnostika GmbH, Mainz, Germany) and an EL808 Ultramicroplate Reader (Bio-Tek Instruments, Winooski, VT, USA).

Urea was measured by a standard laboratory method on Roche Cobas 6000 chemistry analyzer (Roche Diagnostics).

Anti-Xa activity was measured by a chromogenic method (Biophen Heparin, Hyphen BioMed, Neuville-sur-Oise, France) on a STA-C (Diagnostica Stago, Asnières, France) with detection limit 0.05 IU/mL.

Analysis of antithrombin was carried out using a chromogenic method (Coamatic Antithrombin, Chromogenix, Instrumentation Laboratory, Milan, Italy).

Automated measurement of thrombin generation was performed by Calibrated Automated Thrombography (Thrombinoscope, Maastricht, The Netherlands), as reported previously [3]. Thrombin generation was triggered in platelet-poor plasma in the presence of 5 pM tissue factor and a concentration of 4 μM phospholipids. Measurements were performed in duplicate and reported as endogenous thrombin potential (ETP), being the area under the curve of thrombin generation over time. Values were normalized by dividing the patient sample result by the result of a pooled plasma from 50 healthy donors analyzed in the same run.

### Other parameters

Transmembrane pressure (TMP), prefilter pressure, pressure in inlet and outlet bloodline as well as hemoconcentration measured by blood volume sensor (BVS) were registered at 10, 30, 60, 90, 120, 150, 180, 210 and 240 min.

At the end of the session, immediately after blood restitution, visual clotting score of membrane, blood lines, expansion chamber and bubble trap were rated by two unblinded investigators (AD and RP). They rated independently, followed by consensus. The clotting score of the membrane was as follows: 0 = no clotting, 1 = a few colored fibers, 2 = less than 50% of the visible fibers colored and 3 = more than 50% of fibers colored. Clotting of blood lines, expansion chamber and bubble trap was scored as: 0 = no clotting, 1 = discoloration, 2 = minimal clot, 3 = major clot.

Applied compression time was registered.

### Calculations and statistics

Sample size was set at 14, based on feasibility. No prior data on anti-Xa activity in this setting were available to calculate sample size.

The sequence of the sessions per patient was randomized by Quick Calcs (Graphpad software), an online computer system which generates a random number sequence based on the number of experimental groups. The allocation was not concealed.

Reduction ratio (RR), concentration at different time points (Ct after 10, 180 and 240 min) versus the start of dialysis (C0) expressed in %, was calculated as:

$$\text{RR }=\;100\text{ X}\;\left[\left(C_{0}\;–\;C_{t}\right)/C_{0}\right]$$ (1)

Concentrations of beta2microglobulin were corrected for hemoconcentration as measured by BVS:

$$C_{t}{}_{corrected}=C_{t}\text{X }100/\left(100-\text{BVS}\right)$$ (2)

Delta TMP was calculated as the measured TMP at 240 min minus the TMP measured at 10 min. Data are expressed as median and interquartile ranges. Statistics and figures were generated with SPSS (SPSS Inc., Chicago, IL) and GraphPad Prism 4.0 (GraphPad Software, San Diego, California). Continuous paired data were analyzed with repeated measures analysis of variance (Friedman) followed by Wilcoxon in case of significance. Chi square test was performed for categorical variables. Correlations were tested with Spearman correlation test. Receiver Operator Characteristic (ROC) analysis was applied to determine cut off values of anti-Xa. Significance was accepted if p<0.05.

### Deviation from initial protocol

Initially we intended to measure clearances for urea and beta2microglobulin as measure of dialysis efficiency. This was however not feasible due to the important interference of the decrease in bloodwaterflow due to ultrafiltration. Hence dialysis efficiency was expressed as reduction ratio of urea and beta2microglobulin.

Primary and secondary end-points were initially ill-defined; a shift towards secondary end-points with only one primary end-point left was made.

Patient N° 5 received his second experimental session (IN_0_) 2 days later than planned because of a technical problem.

Initially we intended to have 14 patients completing the study. Due to feasibility issues we did not replace patient N° 4 (after his exclusion from the study due to infection). So we ended up with 13 patients completing the study.

## Results

The median and interquartile range of the administered dose of tinzaparin was 66.0 (54.5–69.8) IU/kg body weight (S1 Fig). No premature interruptions of the sessions or bleeding occurred.

In Table 1, predialysis values of hematocrit, antithrombin and CRP are shown as well as ultrafiltration and substitution volumes and fistula compression times. No differences between the 3 schedules were observed.

**Table 1:** Predialysis hematocrit, antithrombine, CRP and applied volumes and compression times: median and interquartile range.

	IN_0_	IN_5_	OUT_0_
Hematocrit (%)	36 (35–39)	36 (35–39)	37 (34–38)
Antithrombin (%)	97 (92–112)	97 (86–99)	93 (86–113)
CRP (mg/L)	3 (1–7)	3 (2–6)	2 (2–11)
Ultrafiltered volume (L)	1.65 (0.79–1.88)	1.69 (0.86–2.42)	1.39 (0.79–2.38)
Substitution volume (L)	15.8 (15.2–16.3)	15.7 (14.8–16.4)	16.1 (15.6–16.7)
Applied compression time inlet needle (min)	16 (13–18)	15 (13–16)	15 (9–18)
Applied compression time outlet needle (min)	14 (9–16)	12 (9–16)	14 (7–14)

Abbreviations: IN_0_: tinzaparin injection before the start of the session at the inlet blood line; IN_5_: injection 5 min after the start of the session at the inlet blood line and OUT_0_: injection before the start of the session at the outlet blood line; C Reactive Protein (CRP.)

Comparison between schedules: no significant differences.

### Coagulation parameters

#### Anti-Xa activity

The evolution of anti-Xa activity is displayed in Table 2 and S2 Fig Predialysis anti-Xa activity was consistently zero. The highest values were measured at 30 min, followed by a gradual decrease. When comparing the different schedules, the lowest anti-Xa activity was measured during the sessions with administration of tinzaparin at the inlet blood line before the start (IN_0_). No differences in anti-Xa levels were observed between IN_5_ and OUT_0_.

**Table 2:** Anti-Xa activity: median and interquartile range.

	IN_0_	IN_5_	OUT_0_	Comparison between schedules: *p*
Pre	0 (0–0)	0 (0–0)	0 (0–0)	
30 min	0.95 (0.88–1.27)	1.13 (1.06–1.33)	1.12 (0.89–1.37)	IN_0_ vs IN_5_:0.01IN_0_ vs OUT_0_:0.094 IN_5_ vs OUT_0_:0.37
120 min	0.65 (0.46–0.97)	0.77 (0.56–0.97)	0.74 (0.57–1.07)	IN_0_ vs IN_5_:<0.001IN0 vs OUT_0_:0.003IN_5_ vs OUT_0_:0.63
180 min	0.34 (0.25–0.75)	0.50 (0.34–0.78)	0.47 (0.29–0.85)	IN_0_ vs IN_5_:0.01IN_0_ vs OUT_0_:0.01IN_5_ vs OUT_0_:0.34
240 min	0.14 (0.09–0.45)	0.24 (0.17–0.60)	0.25 (0.15–0.64)	IN_0_ vs IN_5_:<0.001IN_0_ vs OUT_0_:<0.001IN_5_ vs OUT_0_:0.45
Comparison between time points: *p*	Between all time points: 0.001	Pre vs 240 min: 0.002Between all other time points: 0.001	Between all time points: 0.001	

Abbreviations: IN_0_: tinzaparin injection before the start of the session at the inlet blood line; IN_5_: injection 5 min after the start of the session at the inlet blood line and OUT_0_: injection before the start of the session at the outlet blood line.

#### Endogenous thrombin potential (ETP)

The evolution of ETP is presented in Table 3. ETP decreased from normal values predialysis to zero at 30 and 120 min. After 180 and 240 min ETP was again detected in 8 and 21 out of 39 sessions, respectively. When tinzaparin was administered at the inlet line before the start (IN_0_), a higher ETP was observed compared to the sessions where tinzaparin was administered after 5 min (IN_5_) or at the outlet line (OUT_0_).

**Table 3:** Endogenous Thrombin Potential (ETP): median and interquartile range.

	IN_0_	IN_5_	OUT_0_	Comparison between schedules: *p*
Pre	0.96 (0.84–1.16)	0.97 (0.88–1.12)	0.97 (0.86–1.14)	IN_0_ vs IN_5_:0.19IN_0_ vs OUT_0_:0.94IN_5_ vs OUT_0_:0.50
30 min	0 (0–0)	0 (0–0)	0 (0–0)	
120 min	0 (0–0)	0 (0–0)	0 (0–0)	
180 min	0 (0–0.07)	0 (0–0)	0 (0–0)	IN_0_ vs IN_5_:0.062IN_0_ vs OUT_0_:0.19IN_5_ vs OUT_0_:1
240 min	0.39 (0–0.60)	0.06 (0–0.17)	0.01 (0–0.09)	IN_0_ vs IN_5_:0.031IN_0_ vs OUT_0_:0.039IN_5_ vs OUT_0_:0.55
Comparison between time points: *p*	Pre vs all time points: <0.00130 and 120 min vs 180 min: 0.04330 and 120 min vs 240 min: 0.018180 vs 240 min: 0.016	Pre vs all time points: <0.00130 and 120 min vs 180 min: 0.1830 and 120 min vs 240 min: 0.018180 vs 240 min: 0.016	Pre vs all time points: <0.00130 and 120 min vs 180 min: 0.3230 and 120 min vs 240 min: 0.018180 vs 240 min: 0.016	

Abbreviations: IN_0_: tinzaparin injection before the start of the session at the inlet blood line; IN_5_: injection 5 min after the start of the session at the inlet blood line and OUT_0_: injection before the start of the session at the outlet blood line.

#### Visual clotting scores

Visual clotting score of blood lines was consistently scored 0 for all sessions. Visual clotting scores of membrane, expansion chamber and bubble trap were not different in the three administration schedules (S3 Fig).

#### Relationship between clotting parameters

**Fig 2:**
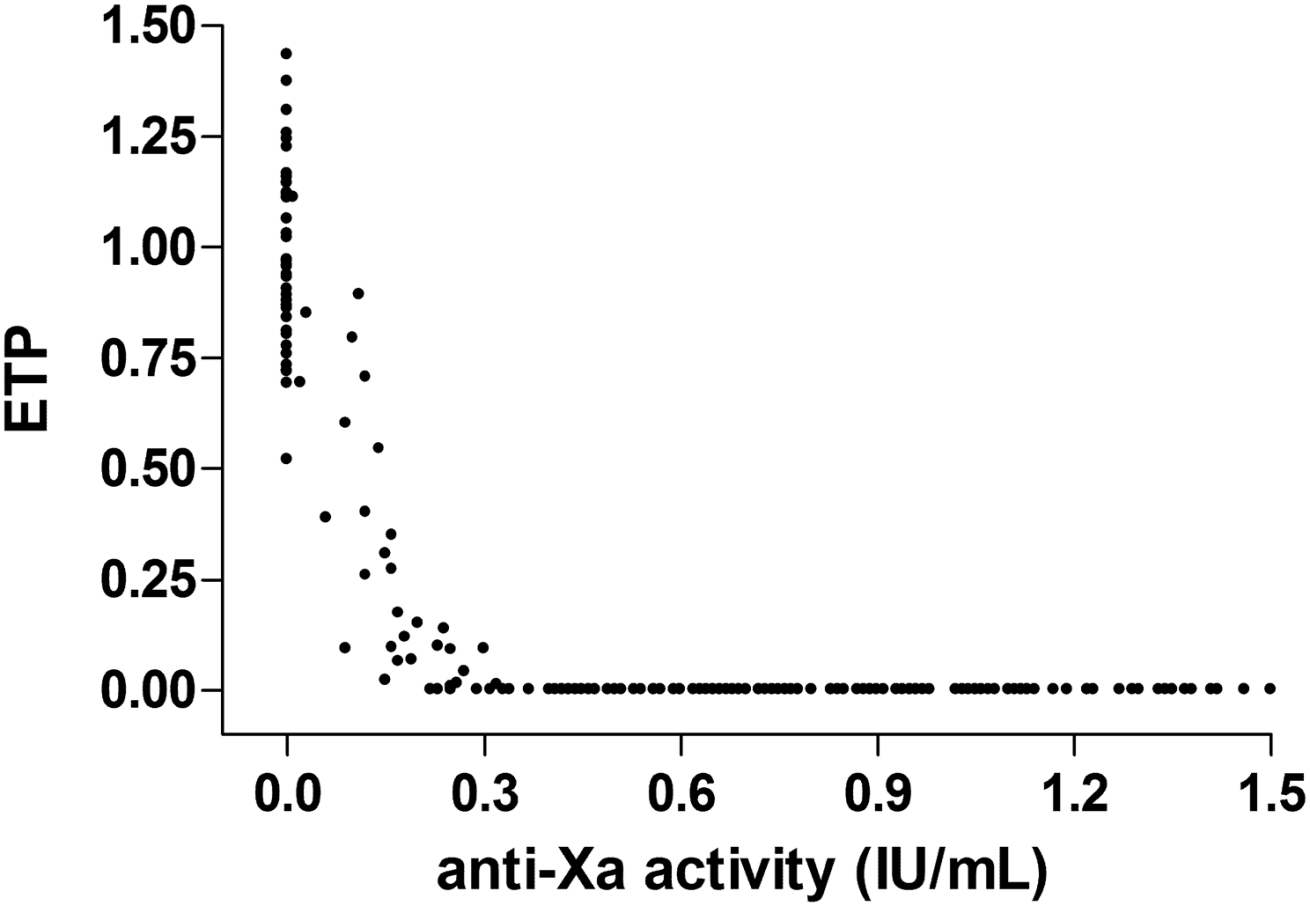
Endogenous thrombin potential (ETP) in relation to anti-Xa activity.

The relation between ETP and anti-Xa activity can be appreciated from Fig 2. When anti-Xa activity is zero, ETP is present. As soon as anti-Xa rises above 0.3 IU/mL, ETP is undetectable. With an anti-Xa value below 0.3 IU/mL, a gradual increase in ETP is observed with decreasing anti-Xa.

Using ROC analysis, an anti-Xa activity of 0.3 IU/mL was able to discriminate for detectable ETP with a sensitivity and specificity of 0.953 and 0.985, respectively (area under the ROC curve of 0.996 with 95% confidence interval: 0.99–1.00).

When evaluating the visual clotting score of the membrane, bubble trap and expansion chamber in function of anti-Xa activity at the end of the session, severe clotting (score 3) was not observed in patients with residual anti-Xa activity above 0.3 IU/mL at the end of the session (S4 Fig). When comparing visual clotting scores in patients with a residual anti-Xa activity below versus above 0.3 IU/mL, a significantly higher clotting score of bubble trap and expansion chamber was observed in those with anti-Xa below 0.3 IU/mL (p<0.001 and p = 0.012, respectively). When comparing visual clotting scores in patients with or without ETP at 240 min, also a higher clotting score of bubble trap and expansion chamber was observed in patients with ETP (p<0.001 and p = 0.014, respectively).

### Pressure measurement

#### Transmembrane pressure (TMP)

TMP increased during the session from 53 (48–57) mmHg at 10 min to 85 (78–104) at 240 min (p<0.001) for IN_0_, from 52 (47–56) to 79 (70–99) for IN_5_ (p<0.001) and from 54 (48–57) to 90 (77–103) mmHg for OUT_0_ (p<0.001). No differences were observed between the three groups, neither at 10 nor at 240 min.

The increase in TMP at the end of the session expressed as delta TMP was not different between the three administration schedules being 37 (31–48), 27 (22–42) and 38 (24–49) mmHg for IN_0_, IN_5_ and OUT_0_, respectively.

**Fig 3:**
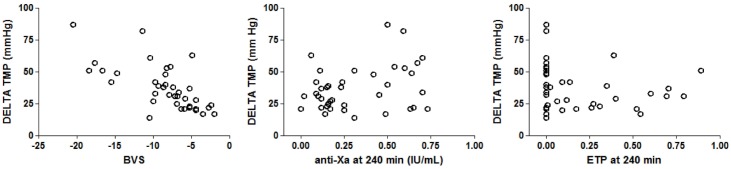
Delta transmembrane pressure (TMP) in relation to hemoconcentration (BVS), anti-Xa-activity and endogenous thrombin potential (ETP). Delta TMP is inversely correlated with hemoconcentration (p<0.0001, Spearman r = -0.68), no significant correlation between delta TMP and anti-Xa level or ETP at 240 min.

Delta TMP was inversely correlated with hemoconcentration (p<0.001, Spearman r = -0.68), whereas no relation was seen with anti-Xa level or ETP at 240 min (Fig 3).

#### Other pressures

No significant differences were observed in inlet or outlet line pressures or in prefilter pressure between the three schedules (S5 Fig).

### Parameters related to dialysis adequacy

#### Reduction ratio (RR)

RR of urea after 240 min was 77.5 (76.0–81.1), 78.9 (73.8–81.9) and 77.5 (73.6–85.1), with IN_0_, IN_5_ and OUT_0_ respectively, being slightly lower with IN_0_ compared to IN_5_ (p = 0.013). For the other time points no differences were noted between the three schedules (S1 Table).

RR of beta2microglobulin after 240 min was 82.1 (74.2–84.9), 80.8 (76.5–84.7) and 81.7 (75.9–85.6) with IN_0_, IN_5_ and OUT_0_ respectively. No differences in RR of beta2microglobulin between the schedules were observed (S1 Table).

#### Relation between clotting parameters and dialysis efficiency

**Fig 4:**
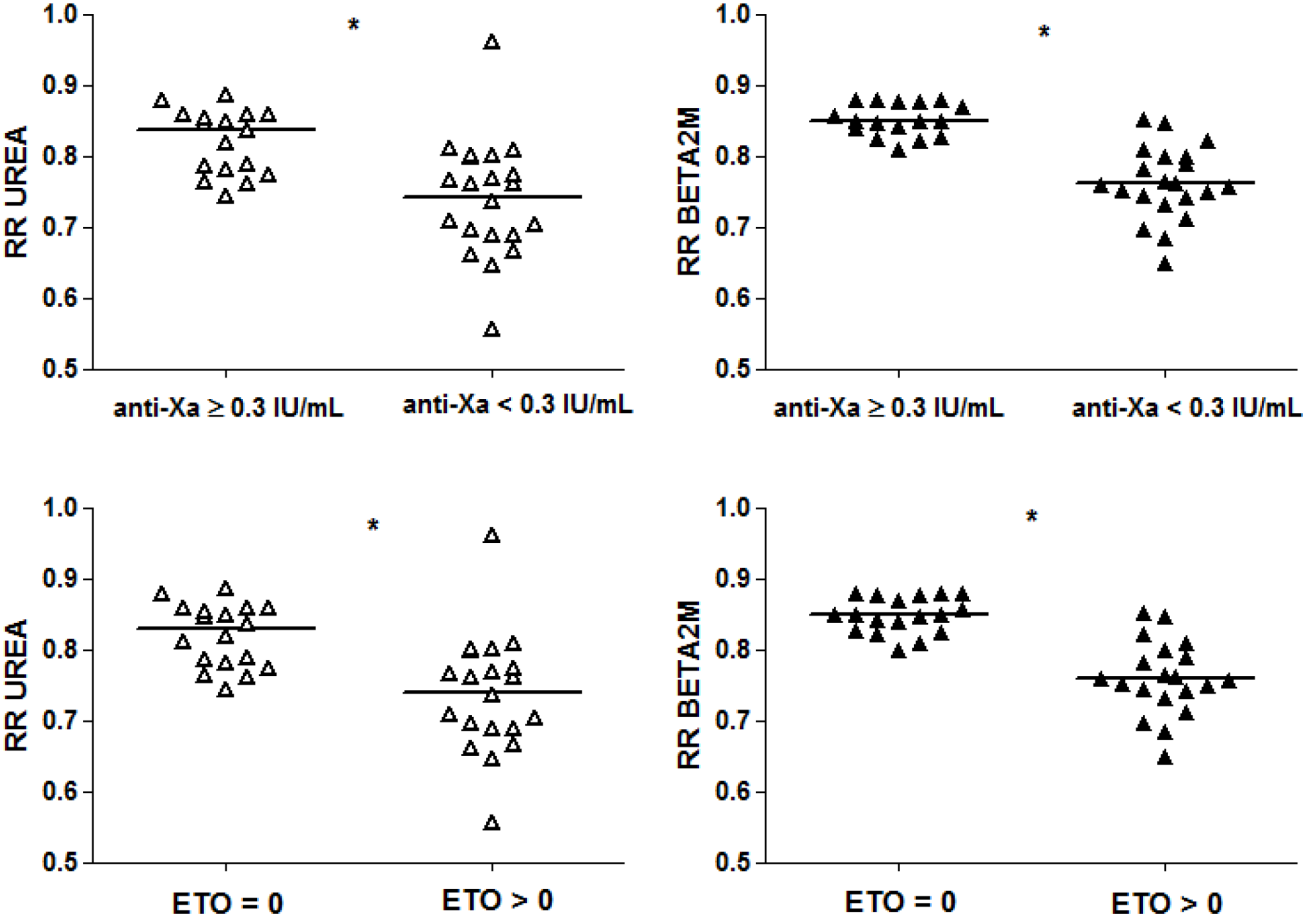
Reduction ratio of urea and beta2microglobulin in relation to anti-Xa activity and ETP. Reduction ratio (RR) of urea (unfilled triangles) and beta2microglobulin (BETA2M) (filled triangles) in sessions with anti-Xa activity above or equal vs. below 0.3 IU/mL and detectable (ETP > 0) vs undetectable ETP (ETP = 0) at 240 min. Comparison between groups: * p<0.001.

As illustrated in Fig 4, RR of urea and beta2microglobulin at 240 min were lower in sessions with an anti-Xa activity at 240 min below 0.3 IU/mL vs. equal or above 0.3 IU/mL: 0.76 (0.69–0.80) vs. 0.84 (0.78–0.86) and 0.76 (0.74–0.80) vs. 0.85 (0.84–0.88), respectively (p<0.001).

RR of urea and beta2microglobulin were also lower in patients with detectable ETP compared to patients with undetectable ETP at the end of the session: 0.75 (0.69–0.78) vs. 0.83 (0.78–0.86) and 0.76 (0.74–0.79) vs. 0.85 (0.83–0.87), respectively (p<0.001).

No relation was observed between visual clotting score and RR of neither urea nor beta2microglobulin.

## Discussion

In the present study we found that administration of tinzaparin at the inlet blood line before the start of the session (IN_0_) resulted in lower anti-Xa activity and higher thrombin generation compared to administration at the outlet line (OUT_0_) or administration 5 min after start of the session (IN_5_). Anti-Xa levels below 0.3 IU/mL at the end of postdilution hemodiafiltration sessions were associated with more thrombin generation, worse visual clotting score and lower RR for urea and beta2microglobulin.

Although advised by experts [2, 4, 5], administration at the outlet line is, at least based on literature data, rarely applied [6, 7]. In most studies LMWHs are injected at the inlet line [8–20] or the administration site is not specified [21–24]. In the information leaflet of tinzaparin, enoxaparin and nadroparin, administration via the arterial line is recommended [25], whereas in guidelines no recommendation concerning the administration site is provided [1].

However, in spite of the currently frequent use of high-flux membranes and hemodiafiltration, the effect of administration route of LMWHs has, to the best of our knowledge, never been studied systematically. With low-flux membranes, Vanuytsel et al. found no differences in anti-Xa activity after administration of nadroparin either at the inlet or outlet line [26].

Tinzaparin has compared to other LMWHs the longest chains. Hence, it is very likely that when using LMWHs with shorter chains such as enoxaparin and nadroparin, removal will likewise occur or even be more pronounced. The removal of enoxaparin during hemodiafiltration has previously been demonstrated, however without assessing the impact of the administration site [27].

We ascribe the lower anti-Xa observed in the IN_0_ schedule, to dialytic/convective removal of unbound tinzaparin from the rinsing solution through a yet uncoated dialyzer membrane. Also enhanced adsorption to the uncoated membrane could play a role. Alternatively, the lower anti-Xa levels with IN_0_ could simply be the logical consequence of 5 min earlier administration compared to the IN_5_ schedule. A simulation based on kinetic parameters, however resulted in a difference of barely 0.01 IU/mL anti-Xa activity at the end of dialysis with 5 min difference in administration time, whereas the difference that was observed here was in the range of 0.1 IU/mL. Hence the role of such small difference in time as applied here is probably negligible.

In addition to anti-Xa, thrombin generation was assessed by ETP determination. Thrombin generation assays measure the ability of plasma to generate thrombin following ex vivo activation of coagulation with tissue factor [28]. Hence, not only the initiation phase, but also the propagation and terminal phase of clotting are measured; ETP reflects the potential thrombin forming capacity. As the ultimate goal of anticoagulation during dialysis is the prevention of clot formation, measurement of ETP is highly informative. We found that anti-Xa activity of 0.3 IU/mL was able to discriminate for measurable ETP. When comparing the different routes of administration, higher ETP was observed with IN_0_ compared to IN_5_ and OUT_0_.

In healthy controls, ETP was found to be a valuable test for measuring the anticoagulant effect of heparins [29]. Only limited data on thrombin generation assays in hemodialysis are available. Predialysis ETP values were found either lower [30] or higher [31] compared to healthy controls. Vernom et al. found a decreased postdialysis ETP compared to predialysis values [6]. In contrast to our data, however, they consistently demonstrated thrombin generation in postdialysis samples. This could be attributed to the lower tinzaparin doses used in their patients, compared to ours.

In addition to coagulation parameters, we also evaluated dialysis performance by calculating the RR of urea and beta2microglobulin. If fibers occlude, it is expected that RR of uremic retention solutes will be lower. In the present study however, no clinically important differences in RR were observed between the three administration schedules.

Interestingly, but initially not defined as study outcome, we noticed lower RR of both urea and beta2microglobulin in sessions ending with an anti-Xa activity below 0.3 IU/mL. Also in sessions with detectable ETP, a lower RR of these uremic retention solutes was observed. To the best of our knowledge we are the first to study the anticoagulant effect of LMWH during hemodiafiltration by measuring RR in relation to anti-Xa and ETP.

No differences in visual clotting score were noted between the three administration schedules. A limitation of the present study is the fact that the investigators rating the visual clotting score were not blinded to the injection mode.

In conclusion, the injection of tinzaparin, and by extrapolation probably of all LMWHs with a MW smaller than or equal to that of tinzaparin, at the blood inlet line before the start of hemodiafiltration is associated with loss of anticoagulant activity. This procedure although widely applied cannot be recommended as it associated with washing away of expensive medication. Injection of LMWH at the outlet line or in the inlet line after 5 min are better alternatives.

In postdilution volume controlled hemodiafiltration, with an exchange volume of 25% of blood flow and anticoagulated with tinzaparin, an anti-Xa activity below 0.3 IU/mL at the end of a 4 hour session, is associated with thrombin generation, a higher clotting score and a decreased RR of urea and beta2microglobulin. Based on these results, it can be hypothesized that if tinzaparin dose is targeted to a level slightly above 0.3 IU/mL at the end of the session, not only improved dialysis efficiency is obtained but in addition overdosing of LMWH will be avoided. These data need however confirmation from a larger sample sized study in which patients are injected with various doses of tinzaparin, in order to obtain anti-Xa activity around 0.3 IU/ml at the end of the session.

## Supporting Information

S1 Fig Tinzaparin dose in relation to body weight and anti-Xa activity in relation to dose per kg body weight. (TIF)

S2 Fig Median anti-Xa activity (IU/mL) measured at different time points. Full bars: IN_0_, open bars: IN5 and hatched bars: OUT_0_. *: p<0.05 vs. IN_0_.
(TIF)

S3 Fig Visual clotting scores of membrane, bubble trap and expansion chamber. (TIF)

S4 Fig Visual clotting scores of membrane, bubble trap and expansion chamber in relation to anti-Xa activity (IU/mL) measured at 240 min. (TIF)

S5 Fig Evolution of pressure during the sessions. Open squares and full lines: IN_0_, full squares and datched lines: IN_5_, open triangles and full lines: OUT_0_.
(TIF)

S1 Table Reduction ratio (RR) of urea and beta2microglobulin (Beta2M): median and interquartile range. (DOCX)

S1 Text Protocol English version. (DOC)

S2 Text Protocol Dutch version. (DOC)

S3 Text CONSORT checklist. (DOC)
