# Supplementary material for: Where and When To Inject Low Molecular Weight Heparin in Hemodiafiltration? A Cross Over Randomised Trial
Source: PLoS One. 2015 Jun 15;10(6):e0128634. doi: 10.1371/journal.pone.0128634 (PMC4468116; doi:10.1371/journal.pone.0128634)
Supplement: S1 Table — (DOCX) [file pone.0128634.s006.docx]

Supplemental Table: Reduction ratio (RR) of urea and beta2microglobulin (Beta2M): median and interquartile range

| RR urea | IN_0_ | IN_5_ | OUT_0_ |
| --- | --- | --- | --- |
| 10 min | 18.3 (16.6-19.6) | 18.8 (15.6-20.3) | 17.4 (15.9-20.0) |
| 180 min | 69.7 (63.8-75.5) | 69.7 (63.2-74.3) | 68.7 (62.9-72.1) |
| 240 min | 77.5 (76.0-81.1) | 78.9 (73.8-81.9) ^*^ | 77.5 (73.6-85.1) |
|  |  |  |  |
| RR Beta2M | IN_0_ | IN_5_ | OUT_0_ |
| 10 min | 29.6 (25.2-40.8) | 32.6 (30.8-37.0) | 40.3 (24.1-44.8) |
| 180 min | 77.7 (67.1-82.2) | 79.9 (70.1-82.0) | 78.2 (70.4-82.6) |
| 240 min | 82.1 (74.2-84.9) | 80.8 (76.5-84.7) | 81.7 (75.9-85.6) |

Abbreviations: IN_0_: tinzaparin injection before the start of the session at the inlet blood line; IN_5_: injection 5 min after the start of the session at the inlet blood line and OUT_0_: injection before the start of the session at the outlet blood line.

Comparison between schedules ^*^: p<0.05 vs. IN_0_
