# Supplementary material for: Where and When To Inject Low Molecular Weight Heparin in Hemodiafiltration? A Cross Over Randomised Trial
Source: PLoS One. 2015 Jun 15;10(6):e0128634. doi: 10.1371/journal.pone.0128634 (PMC4468116; doi:10.1371/journal.pone.0128634)
Supplement: S1 Text — (DOC) [file pone.0128634.s007.doc]

# Part I : Study-related part of the protocol

# Title of the study

The use of low molecular weight heparin (LMGH) during hemodialysis.

# Number of the trial

Protocol: AGO/2008/010

EudraCT number: 2008-005224-91

# Objectives of the study

The research questions are:

1) Is clotting better prevented if LMWH are injected early in the session?

2) Does the injection of LMWH at the outlet line instead of the inlet line results in different aXa levels and does this has an influence on clotting?

3) Does clotting and/or aXa activity has an influence on the clearance of small and/or larger molecules during dialysis?

# General information

## Researcher(s)

Prof. dr. Annemieke Dhondt

Dienst Nierziekten

De Pintelaan 185

9000 Gent

[Annemie.dhondt@ugent.be](mailto:Annemie.dhondt@ugent.be)

09/332 45 79

Prof. dr. Raymond Vanholder

Dienst Nierziekten

De Pintelaan 185

9000 Gent

[raymond.vanholder@ugent.be](mailto:raymond.vanholder@ugent.be)

09/332 45 22

## Sponsor

Universitair Ziekenhuis Gent

## Departments/laboratories involved in study

Dienst Nierziekten

Universitair Ziekenhuis Gent

De Pintelaan 185

9000 Gent

Dienst Klinische Biologie

Universitair Ziekenhuis Gent

De Pintelaan 185

9000 Gent

# Introduction

During hemodialysis blood of the patient is taken extracorporeal. Via the inlet blood line, blood is pumped towards the artificial kidney; the cleared blood streams back via the outlet blood line to the patient. Bringing the blood extracorporeal leads to activation of the clotting cascade. This can lead to clotting with complete occlusion of the circuit resulting in bloodloss or to more subtle degrees of clotting: 1/ A number of capillaries of the artificial kidney can occlude. This can result in a decreased dialysis efficiency and an increase in prefilter pressure. 2) A number of pores of the artificial membrane can occlude, resulting in a decreased dialysis efficiency and an increase in transmembrane pressure. 3) Pore diameter can decrease, resulting in a decrease in removal of the large molecules, while removal of small molecules is maintained.

To avoid clotting in the extracorporeal circuit, low molecular weight heparins (LMWH) in prefilled syringes are used in our center. The injection is routinely done at the inlet blood line after the start of dialysis. A direct intravenous injection in the arterio-venous fistula is not possible due to the presence of a non removable injection needle. Potential problems associated with this procedure are that the LMWH are administered too late and that clotting is initiated already eventually leading to loss of dialysis efficiency. Another potential problem is that LMWH can be removed by dialysis, especially when LMWH are injected in the inlet blood line, before the start. In the absence of blood in the circuit, LMWH cannot bind to proteins. As the molecular weight of LMWH is on average 4.5 kD, these molecules can, if unbound, be removed by highflux membranes. Highflux membranes have large pores, they are used in high flux hemodialysis and hemodiafiltration.

# Present study

## Project outline

Cross-over, prospective, randomised, open

## Medication

### Composition en dose

Innohep 2.500 I.E/0,25 ml - Tinzaparinum natricum 2.500 I.E anti Xa

Innohep 3.500 I.E/0,35 ml - Tinzaparinum natricum 3.500 I.E anti Xa

Innohep 4.500 I.E/0,45 ml - Tinzaparinum natricum 4.500 I.E anti Xa

At the start of hemodialysis one single bolus of 3.500, 4.500 of 6.000 IE is administered at the hemodialysis circuit

### Registrationholder - Distribution

LEO Pharma N.V.

Sneeuwbeslaan 20

2610 Wilrijk

### Distribution

Pharmacy of University Hospital Ghent

### Package

Commercially available package.

### Administration

Three different administration modes of LMWH will be compared:

1) Injection at the inlet blood line just before the start.

2) Injection at the inlet blood line 5 min after the start.

3) Injection at the outlet blood line just before the start.

### Labelling

Commercial available labels.

### Storage

Room temperature

### Known side effects of the drug

Bleeding, thrombocytopenia, allergic reaction

### Drug accountability

Delivery and handling of the products will be documented.

## The test persons

### Number of test persons

### Number of test persons

20 hemodialysis/hemodiafiltration patients

### Inclusion criteria

Older than 18 years

Chronic kidney disease (CKD) stade 5 treated with chronic hemodiafiltration/hemodialysis

Hematocrit above 30%

Signing informed consent

### Exclusion criteria

Treatment with vitamin K antagonists

Treatment with heparin besides when used during dialysis

Thrombocytopenia below 120.000 /µl

Active bleeding

Allergy to heparin

### Replacement of test persons

14 patients should complete the study.

The first 6 patients dropping out the study, will not be replaced, the following will be replaced.

### Limitations and interdictions of test persons

None

### Potential benefits and risks for test persons

With a better prevention of clotting, the dose of LMWH can possibly be reduced and/or the dialysis efficiency will improve.

# Procedures

## Procedures

The following parameters will be measured: Clearance of urea: 10 min after start dialysis en at the end, clearance Beta 2 microglobuline: 10 min after start and at the end, clearance LMGH: 30 min after start, transmembrane pressure, prefilter pressure, inlet line pressure, outlet line pressure.

Clearance will be calculated from concentration in inlet bloodline (Ci) and outlet bloodline (Co), with following formula:

Clearance= blood flow X (Ci-Co)/Ci

Visual inspection after restitution: presence of thrombi, antithrombin at the start, CRP at the start, hematocrit at the start, aXa at 30,120,180,240 min, thrombin generation: pre, after 30,120,180,240 min

Following parameters were also noted: effective dialysis time (minutes), ultrafiltered volume, treated blood volume, blood flow, substitution volume, body weight before and after dialysis, compression time (minutes).

## Study flow

Each patient will be studied in 3 sessions. The three different methods of administration will be compared to each other.

1. Injection at the inlet blood line before start.
2. Injection at the inlet blood line 5 minutes after the start of the blood pump.
3. Injection at the outlet bloodline before start. in de afvoerende bloedlijn vóór aansluiten.

The dose of LMWH remains the same, and is identical to the dose the patient received prior the study to anticoagulate the circuit.

The three methods are assigned to 20 patients in random order. For each patient the study lasts 3 weeks; de sessions under study for the individual patient take place on the same day of the week. Hemodiafiltration is performed. De membrane is FX800 (Fresenius), the dialyse monitor is AKA 200 S. The amount of of ultrafiltration and substitution as well as QB and QD remain for each individual patients constant.

The expected duration of the study is 1 year.

# Randomisation / blinding

Randomisation list is made by Quickcals (Graphpad software). There is no blinding.

# Previous and concommitant medication

The following medication may not be used during the study: vitamine K antagonists, other heparin than during dialysis, urokinase (and other thromboytica).

All other medication is allowed.

Dose of anti platelet medication may not be changed.

# Adverse event reporting

List of abbreviations

AE Adverse Event

CA Competent Authority

EC Ethics Committee

SAE Serious Adverse Event

SSAR Suspected Serious Adverse Reaction

SUSAR Suspected Unexpected Serious Adverse Reaction

Adverse events (AE)

The following information will be recorded:

- nature of adverse event
- date and time of occurrence and disappearance
- intensity: mild, moderate or severe
- frequency: once, continuous or intermittent
- decision regarding study: continuation or withdrawal
- relation to the study medication (see below)

AEs will be recorded from the first drug administration until the end of the trial.

Special attention will be given to those subjects who have discontinued the trial for an AE, or who experienced a severe or a serious AE.

Definitions of Adverse Event (AE)

Any untoward medical occurrence in a patient or clinical investigation subject administered a pharmaceutical product and which does not necessarily have a causal relationship with this treatment. An adverse event (AE) can therefore be any unfavorable and unintended sign (including an abnormal finding), symptom, or disease temporally associated with the use of a medicinal (investigational) product, whether or not related to the medicinal (investigational) product.

*Serious adverse event*

Any untoward medical occurrence that at any dose:

- results in death

- is life-threatening

- requires inpatient hospitalization or prolongation of existing hospitalization,

- results in persistent or significant disability/incapacity,

or

- is a congenital anomaly/birth defect.

Note: Medical and scientific judgement should be exercised in deciding whether expedited reporting is appropriate in other situations, such as important medical events that may not be immediately life-threatening or result in death or hospitalization but may jeopardize the subject or may require intervention to prevent one of the outcomes listed in the definition above.

*Unexpected adverse event*

An adverse event, the nature or severity of which is not consistent with the applicable product information (e.g., Investigator's Brochure for an unapproved investigational product or package insert/summary of product characteristics for an approved product).

*Life-threatening*

Any event in which the subject was at risk of death at the time of the event; it does not refer to an event which hypothetically might have caused death if it were more severe.

*Associated with the use of the drug*

An adverse event is considered associated with the use of the drug if the attribution is possible, probable or very likely.

Attribution definitions

# *Not related*

An adverse event which is not related to the use of the drug.

*Doubtful*

An adverse event for which an alternative explanation is more likely - e.g. concomitant drug(s), concomitant disease(s), and/or the relationship in time suggests that a causal relationship is unlikely.

*Possible*

An adverse event which might be due to the use of the drug. An alternative explanation - e.g. concomitant drug(s), concomitant disease(s), - is inconclusive. The relationship in time is reasonable; therefore the causal relationship cannot be excluded.

*Probable*

An adverse event which might be due to the use of the drug. The relationship in time is suggestive (e.g. confirmed by dechallenge). An alternative explanation is less likely - e.g. concomitant drug(s), concomitant disease(s).

*Very likely*

An adverse event which is listed as a possible adverse reaction and cannot be reasonably explained by an alternative explanation - e.g. concomitant drug(s), concomitant disease(s). The relationship in time is very suggestive (e.g. it is confirmed by dechallenge and rechallenge).

Reporting of adverse events

Adverse events will be reported between the first dose administration of trial medication and the last trial related activity.

Medical events that occur between signing of the Informed Consent and the first intake of trial medication will be documented on the medical and surgical history section and concomitant diseases page of the CRF.

SAEs occurring within a period of 30 days following the last intake of trial medication will also be handled as such if spontaneously reported to the investigator.

The cause of death of a subject in a clinical trial, whether the event is expected or associated with the investigational agent, is a SAE.

All serious adverse events (SAE) and pregnancies occurring during clinical trials must be reported by the local Principal Investigator within 2 working days to:

- The local EC
- The Trial Bureau of the University Hospital Ghent
- The National Coordinating Investigator (in case of multicentre trials)

This reporting is done by using the appropriate SAE form. For the contact details, see below.

It is the responsibility of the local Principal Investigator to report the SAE’s to the local EC(s).

It is the responsibility of the Trial Bureau to report the SUSAR’s to the EC(s) and CA’s according to national legislation

In case the investigator decides the SAE is a SUSAR (Suspected Unexpected Serious Adverse Reaction), the Trial Bureau will report the SUSAR to the Central EC and the CA within the timelines as defined in national legislation. The National Coordinating Investigator reports the SUSAR to all local Principal Investigators.

The first report of a serious adverse event may be made by telephone, e-mail or facsimile (FAX).

Contact details of the Trial Bureau:

e-mail: [Trialbureau@uzgent.be](mailto:Trialbureau@uzgent.be)

tel.: 09/332 89 99

fax: 09/332 89 90

Contact details of the National Coordinating Investigator:

e-mail: [Annemie.dhondt@ugent.be](mailto:Annemie.dhondt@ugent.be)

tel.: 09/332 45 79

fax: 09/332 45 99

The investigator must provide the minimal information: i.e. trial number, subject's initials and date of birth, medication code number, period of intake, nature of the adverse event and investigator's attribution.

This report of a serious adverse event by telephone must always be confirmed by a written, more detailed report. For this purpose the appropriate SAE form will be used. Pregnancies occurring during clinical trials are considered immediately reportable events. They must be reported as soon as possible using the same SAE form. The outcome of the pregnancy must also be reported.

**If the subjects are not under 24-hour supervision of the investigator or his/her staff (out-patients, volunteers), they (or their designee, if appropriate) must be provided with a "trial card" indicating the name of the investigational product, the trial number, the investigator's name and a 24-hour emergency contact number.**

Annual Safety Reporting

The Trial Bureau will ask the National Coordinating Investigator for an annual report containing an overview of all SSARs (Suspected Serious Adverse Reaction) and a summary regarding the safety of the trial subjects. The Trial Bureau will send this report to the Central EC and the CA within the timelines as defined in national legislation. The National Coordinating Investigator will pass this annual report to all local Principal Investigators.

# Study-analysis

## Sample size calculation

The decision to investigate 20 patients is based on availability of patients complying with inclusion criteria and feasibility.

## Analysis of samples

For the measurement of **antitrombine** (AT), citrated plasma will be incubated with an excess of factor X (FXa) in the presence of heparin. Fxa complexes with heparin and AT. The unbound Fxa mediates the hydrolysis of S-2765, a chromogenic substrate, to pNA. The release of pNA is measured at 405 nm. The generation of pNA is inversely proportional with AT activity in plasma.

Quantitative determination of **low molecular weight heparin** (LMWH) in citrated plasma is based on the fact that LMWHs complex with AT, leading to an increase

in anti-FXa-activity of AT. Fxa is added in excess to a mixture of undiluted plasma and the chromogenic S-2732. The concentration of nPA is inversely proportional to LMWH/heparin/other in the sample.

**Thrombingenertion tests** are perfomed with the calibrated Automated Thrombinography-methode. The total amount of generated thrombin is measured. The measurement is done in platelet poor plasma after addition of tissue factor and phospholipids

**CRP** analysis is done with an particle-enhanced imunoturbidimetric assay.

Anti-CRP antibodies are linked to latex particles. These antibodies react with CRP present in the sample generating antibody-antigen complexes, leading to agglutination of the latex particles. The agglutination leads to turbidity in the sample and is measured by turbimetry.

In EDTA blood, the number of red blood cells is counted (impedance). The hematocrit is measured by cumulation of obtained pulses during counting red blood cells.

**Urea** is measured by standard methods, namely enzymatic reaction followed by spectrophotometric analysis.

**Beta2** **microglobulin**: is quantified with wordt gekwantificeerd met een a sandwich ELISA (firma Orgentec Diagnostika GmbH, Mainz, Germany)

Urea en beta2 microglobulin will be measured in the laboratory of nephrology UZ Gent (prof dr R Vanholder).

All other analysis are performed in the Laboratorium voor Klinische Biologie UZ Gent, Speciale stolling en Hematologie (Dr. K. Devreese) en Chemie (Dr. J. Delanghe).

## Statistical analysis

Descriptive statistics.

Repeated measures analysis of variance (Friedman). When significant followed by Wilcoxon signed rank test. Significance is accepted if p<0.05.

The statistical analysis will be performed by Annemieke Dhondt, Dienst nierziekten, UZ Gent.

# Quality control and monitoring

Quality control of data in CRF will be performed by comparing data from source documents and CRF by a person, not involved in entering data in CRF.

# Insurance and compensation

Polis UZ Gent - strictly liability

# Publication

We intend to publish the data in a A1 journal. The privacy of the patients will always be respected.

# Part II : General part of the protocol

# Independent Ethics Committee (IEC) / Institutional Review Bord (IRB)

This trial can only be undertaken after full approval of the protocol and addenda has been obtained from the IEC/IRB. This document must be dated and clearly identify the protocol, amendments (if any), the informed consent form and any applicable recruiting materials and subject compensation programs approved.

During the trial, the following documents will be sent to the IEC/IRB for their review:

- reports of adverse events that are serious, unexpected and associated with the investigational drug
- all protocol amendments and revised informed consent form (if any).

Amendments should not be implemented without prior review and documented approval / favorable opinion form the IEC/IRB except when necessary to eliminate an immediate hazard to trial subjects or when the change involves only logistical or administrative aspects of the trial.

Reports on, and reviews of the trial and its progress will be submitted to the IEC/IRB by the investigator at intervals stipulated in their guidelines.

At the end of the trial, the investigator will notify the IEC/IRB about the trial completion.

# ICH/GCP guidelines

This trial will be conducted in accordance with the protocol, current ICH-GCP guidelines and applicable law(s).

Good Clinical Practice (GCP) is an international ethical and scientific quality standard for designing, conducting, recording and reporting trials that involve the participation of human subjects. Compliance with this standard provides public assurance that the rights, safety and well-being of trial subjects are protected, consistent with the principles that have their origin in the Declaration of Helsinki, and that the clinical trial data are credible.

# Subject information and informed consent

Prior to entry in the trial, the investigator must explain to potential subjects or their legal representatives the trial and the implication of participation. Subjects will be informed that their participation is voluntary and that they may withdraw consent to participate at any time. Participating subjects will be told that their records may be accessed by competent authorities and by authorized persons without violating the confidentiality of the subject, to the extent permitted by the applicable law(s) and/or regulations. By signing the Informed Consent Form (ICF), the subjects or legally acceptable representatives are authorizing such access.

After this explanation and before entry to the trial, written, dated and signed informed consent should be obtained from the subject or legally acceptable representative. The ICF should be provided in a language sufficiently understood by the subject. Subjects must be given the opportunity to ask questions.

The subject or legally acceptable representative will be given sufficient time to read the ICF and to ask additional questions. After this explanation and before entry to the trial, consent should be appropriately recorded by means of either the subject's or his/her legal representative's dated signature or the signature of an independent witness who certifies the subject's consent in writing. After having obtained the consent, a copy of the ICF must be given to the subject.

In case the subject or legally acceptable representative is unable to read, an impartial witness must attest the informed consent.

Subjects who are unable to comprehend the information provided or pediatric subjects can only be enrolled after consent of a legally acceptable representative.

# Case Report Forms

The source documents are to be completed at the time of the subject’s visit. The CRFs are to be completed within reasonable time after the subject’s visit.

The investigator must verify that all data entries in the CRFs are accurate and correct. If certain information is Not Done, Not Available or Not Applicable, the investigator must enter "N.D." or "N.AV." or "N.AP", respectively in the appropriate space.

# Direct access to source data / documents

The investigator will permit trial-related monitoring, audits, IRB/IEC review, and regulatory inspection(s), providing direct access to source data/documents.

# Data handling and record keeping

The investigator and sponsor specific essential documents will be retained for at least 20 years. At that moment, it will be judged whether it is necessary to retain them for a longer period, according to applicable regulatory or other requirement(s).

# Signature page

*Investigator:*

Name: ________________________________________________

Title: ________________________________________________

Signature: ________________________________________________

Date: ________________________________________________

*Investigator:*

Name: ________________________________________________

Title: ________________________________________________

Signature: ________________________________________________

Date: ________________________________________________
