# Supplementary material for: Where and When To Inject Low Molecular Weight Heparin in Hemodiafiltration? A Cross Over Randomised Trial
Source: PLoS One. 2015 Jun 15;10(6):e0128634. doi: 10.1371/journal.pone.0128634 (PMC4468116; doi:10.1371/journal.pone.0128634)
Supplement: S2 Text — (DOC) [file pone.0128634.s008.doc]

# Deel I : Studie-gerelateerd deel van het protocol

# Titel van het onderzoek

Het gebruik van laag moleculair gewicht heparines (LMGH) tijdens hemodialyse.

# Onderzoeksnummer

Protocol: AGO/2008/010

EudraCT number: 2008-005224-91

# Doelstellingen van het onderzoek

De vraagstelling van de studie is:

1) Kan het vroegtijdiger inspuiten van LMGH stolling beter voorkomen?

2) Resulteert het inspuiten van LMGH in de afvoerende lijn ipv in de aanvoerende lijn in verschillende aXa en beïnvloedt dit stolling?

3) Beïnvloedt stolling en/of de aXa activiteit de klaring van grote en/of kleine moleculen tijdens dialyse?

# Algemene informatie

## Onderzoeker(s)

Prof. dr. Annemieke Dhondt

Dienst Nierziekten

De Pintelaan 185

9000 Gent

[Annemie.dhondt@ugent.be](mailto:Annemie.dhondt@ugent.be)

09/332 45 79

Prof. dr. Raymond Vanholder

Dienst Nierziekten

De Pintelaan 185

9000 Gent

[raymond.vanholder@ugent.be](mailto:raymond.vanholder@ugent.be)

09/332 45 22

## Opdrachtgever

Universitair Ziekenhuis Gent

## Departementen/laboratoria betrokken in het onderzoek

Dienst Nierziekten

Universitair Ziekenhuis Gent

De Pintelaan 185

9000 Gent

Dienst Klinische Biologie

Universitair Ziekenhuis Gent

De Pintelaan 185

9000 Gent

# Inleiding

Tijdens hemodialyse wordt het bloed van de patiënt extracorporeel gebracht. Via de aanvoerende bloedlijn wordt het bloed naar de kunstnier gepompt, het gezuiverde bloed stroomt via de afvoerende bloedlijn terug naar de patiënt. Het extracorporeel brengen van het bloed gaat gepaard met activatie van de stollingscascade. Dit kan leiden tot een volledige klontering van het circuit met bloedverlies tot gevolg of tot meer subtiele vormen van klontering: 1) Een deel van de capillairen van de kunstnier kan verstoppen. Dit geeft aanleiding tot een verminderde efficiëntie en tot het oplopen van de prefilterdruk. 2) Een deel van de poriën kan verstoppen. Dit geeft aanleiding tot een verminderde efficiëntie en tot het verhogen van de transmembraandruk. 3) De poriën kunnen verkleinen in diameter. Dit geeft aanleiding tot verlies van klaring voor de grotere moleculen, terwijl de klaring van de kleinere moleculen intact blijft. Om stolling in het extracorporeel circuit te vermijden wordt in ons centrum laag moleculair gewicht heparine (LMGH) in voorgevulde spuiten gebruikt. De inspuiting gebeurt routinematig in de aanvoerende bloedlijn van het dialysecircuit na de start van de dialyse. Een directe intraveneuze toediening in de arterio-veneuze fistel is onmogelijk door de aanwezigheid van een niet-verwijderbare injectienaald.

De vrees bestaat dat met de huidige werkwijze de LMGH te laat wordt toegediend en dat daardoor stolling en eventueel verlies aan effiëntie van dialyse optreedt. Een tweede potentieel probleem is dat LMGH kan uitgedialyseerd worden. Dit laatste is voornamelijk te vrezen als LMGH wordt ingespoten in de aanvoerende bloedlijn vóór aansluiten. Op dat ogenblik is nog geen bloed met antithrombine aanwezig voor binding van LMGH. Het gemiddeld MG van LMGH is 4.5 kD. Tijdens highflux hemodialyse of hemodiafiltratie (waar membraan met grote poriën wordt gebruikt) kunnen moleculen met dergelijk MG verwijderd worden.

# Het huidig onderzoek

## Onderzoeksplan

cross-over, prospectief, gerandomiseerd, open

## Medicatie

### Samenstelling en dosering

Innohep 2.500 I.E/0,25 ml - Tinzaparinum natricum 2.500 I.E anti Xa

Innohep 3.500 I.E/0,35 ml - Tinzaparinum natricum 3.500 I.E anti Xa

Innohep 4.500 I.E/0,45 ml - Tinzaparinum natricum 4.500 I.E anti Xa

Bij de start van de hemodialyse wordt er een éénmalige bolus van 3.500, 4.500 of 6.000 IE via het hemodialysecircuit toegediend.

### Registratiehouder - Distributeur

LEO Pharma N.V.

Sneeuwbeslaan 20

2610 Wilrijk

### Distributeur

Ziekenhuisapotheek UZ Gent

### Verpakking

Commercieel beschikbare verpakking.

### Toedieningsweg

Drie verschillende methodes van toediening van LMGH zullen met elkaar vergeleken worden:

1) Inspuiting in de aanvoerende bloedlijn bij aansluiten.

2) Inspuiting in de aanvoerende bloedlijn 5 minuten na start bloedpomp

3) inspuiting in de afvoerende bloedlijn vóór aansluiten.

### Etikettering

Commercieel beschikbare etikettering.

### Bewaarcondities

Kamertemperatuur

### Gekende bijwerkingen van het geneesmiddel

Bloedingen, thrombopenie, overgevoeligheidsreacties

### Drug accountability

Levering en hantering van de onderzoeksproducten zal gedocumenteerd worden.

## De proefpersonen

### Aantal proefpersonen

20 hemodialyse/hemodiafiltratie patiënten

### Inclusiecriteria

Leeftijd > 18 jaar

Chronic kidney disease (CKD) stadium 5 waarvoor chronische hemodiafiltratie of hemodialyse

Hematocriet > 30%

Tekenen van informed consent

### Exclusiecriteria

Behandeling met vitamine K antagonisten

Behandeling met andere heparines dan deze gebruikt tijdens dialyse

Thrombopenie < 120.000 /µl

Actieve bloeding

Allergie op heparine

### Vervanging van proefpersonen

14 patiënten moeten de studie voleindigen.

De eerste 6 patiënten die uit de studie vallen, zullen niet vervangen worden, de volgende wel.

### Beperkingen en verboden voor de proefpersonen

Geen

### Mogelijke voordelen en risico’s voor de proefpersonen

Als stolling beter kan worden voorkomen, zal vermoedelijk de dosis LMGH kunnen worden verminderd en/of zal de dialyse efficiëntie toenemen.

# Procedures

## Procedures

Volgende parameters worden gemeten: Klaring ureum: 10 min na start dialyse en vóór afsluiten, klaring Beta 2 microglobuline: 10 min na start en vóór afsluiten, klaring LMGH: 30 min na start, transmembraandruk, prefilterdruk, druk aanvoerende en afvoerende bloedlijn.

Klaring wordt berekend uit concentratie in de aanvoerende bloedlijn (Ci) en afvoerende bloedlijn (Co), met volgende formule:

Klaring= bloeddebiet X (Ci-Co)/Ci

Visuele inspectie na afsluiten: aanwezigheid van klonters, Anti thrombine bij begin studie, CRP bij begin studie, Hematocriet bij begin studie, AXa meting: 30, 120, 180, 240 min, Thrombine generatie: pre, 30, 120, 180, 240 min.

Volgende parameters worden genoteerd: effectieve dialyseduur (minuten), geültrafiltreerd volume, verzet bloedvolume, bloeddebiet, substitutievolume en lichaamsgewicht vóór en na dialyse, afduwtijd fistel(minuten).

## Studieverloop

Elke patiënt wordt gedurende 3 sessies bestudeerd. Drie verschillende methodes van toediening van LMGH zullen met elkaar vergeleken worden.

1. Inspuiting in de aanvoerende bloedlijn bij aansluiten.
2. Inspuiting in de aanvoerende bloedlijn 5 minuten na start bloedpomp
3. Inspuiting in de afvoerende bloedlijn vóór aansluiten.

Er wordt steeds dezelfde dosis LMGH ingespoten, namelijk die dosis die de patiënt routinematig krijgt als anticoagulans tijdens dialyse.

De drie methodes worden in willekeurige volgorde bij 20 patiënten toegepast. De studie duurt voor elke patiënt drie weken. De bestudeerde sessie valt voor elke patiënt op dezelfde dag van de week. Hemodiafiltratie postdilutie wordt toegepast. De gebruikte membraan is FX800 (Fresenius), het gebruikte dialysetoestel is AKA 200 S. De hoeveelheid ultrafiltratie en substitutievocht alsook QB en QD blijft voor elke patiënt constant.

De verwachte duur van de ganse studie bedraagt 1 jaar.

# Randomisatie / blindering

Randomisatielijst wordt gemaakt met behulp van Quickcals (Graphpad software). Er is geen blinding.

# Voorgaande en concomitante therapie

Volgende medicatie mag tijdens de studie niet gebruikt worden: vitamine K antagonisten, andere heparine dan tijdens dialyse, urokinase (en andere klonteroplossende medicatie).

Alle andere medicatie is wel toegestaan.

Medicatie die de plaatsaggregatie beïnvloedt mag niet in dosis gewijzigd worden tijdens de studie.

# Adverse event reporting

List of abbreviations

AE Adverse Event

CA Competent Authority

EC Ethics Committee

SAE Serious Adverse Event

SSAR Suspected Serious Adverse Reaction

SUSAR Suspected Unexpected Serious Adverse Reaction

Adverse events (AE)

The following information will be recorded:

- nature of adverse event
- date and time of occurrence and disappearance
- intensity: mild, moderate or severe
- frequency: once, continuous or intermittent
- decision regarding study: continuation or withdrawal
- relation to the study medication (see below)

AEs will be recorded from the first drug administration until the end of the trial.

Special attention will be given to those subjects who have discontinued the trial for an AE, or who experienced a severe or a serious AE.

Definitions of Adverse Event (AE)

Any untoward medical occurrence in a patient or clinical investigation subject administered a pharmaceutical product and which does not necessarily have a causal relationship with this treatment. An adverse event (AE) can therefore be any unfavorable and unintended sign (including an abnormal finding), symptom, or disease temporally associated with the use of a medicinal (investigational) product, whether or not related to the medicinal (investigational) product.

*Serious adverse event*

Any untoward medical occurrence that at any dose:

- results in death

- is life-threatening

- requires inpatient hospitalization or prolongation of existing hospitalization,

- results in persistent or significant disability/incapacity,

or

- is a congenital anomaly/birth defect.

Note: Medical and scientific judgement should be exercised in deciding whether expedited reporting is appropriate in other situations, such as important medical events that may not be immediately life-threatening or result in death or hospitalization but may jeopardize the subject or may require intervention to prevent one of the outcomes listed in the definition above.

*Unexpected adverse event*

An adverse event, the nature or severity of which is not consistent with the applicable product information (e.g., Investigator's Brochure for an unapproved investigational product or package insert/summary of product characteristics for an approved product).

*Life-threatening*

Any event in which the subject was at risk of death at the time of the event; it does not refer to an event which hypothetically might have caused death if it were more severe.

*Associated with the use of the drug*

An adverse event is considered associated with the use of the drug if the attribution is possible, probable or very likely.

Attribution definitions

# *Not related*

An adverse event which is not related to the use of the drug.

*Doubtful*

An adverse event for which an alternative explanation is more likely - e.g. concomitant drug(s), concomitant disease(s), and/or the relationship in time suggests that a causal relationship is unlikely.

*Possible*

An adverse event which might be due to the use of the drug. An alternative explanation - e.g. concomitant drug(s), concomitant disease(s), - is inconclusive. The relationship in time is reasonable; therefore the causal relationship cannot be excluded.

*Probable*

An adverse event which might be due to the use of the drug. The relationship in time is suggestive (e.g. confirmed by dechallenge). An alternative explanation is less likely - e.g. concomitant drug(s), concomitant disease(s).

*Very likely*

An adverse event which is listed as a possible adverse reaction and cannot be reasonably explained by an alternative explanation - e.g. concomitant drug(s), concomitant disease(s). The relationship in time is very suggestive (e.g. it is confirmed by dechallenge and rechallenge).

Reporting of adverse events

Adverse events will be reported between the first dose administration of trial medication and the last trial related activity.

Medical events that occur between signing of the Informed Consent and the first intake of trial medication will be documented on the medical and surgical history section and concomitant diseases page of the CRF.

SAEs occurring within a period of 30 days following the last intake of trial medication will also be handled as such if spontaneously reported to the investigator.

The cause of death of a subject in a clinical trial, whether the event is expected or associated with the investigational agent, is a SAE.

All serious adverse events (SAE) and pregnancies occurring during clinical trials must be reported by the local Principal Investigator within 2 working days to:

- The local EC
- The Trial Bureau of the University Hospital Ghent
- The National Coordinating Investigator (in case of multicentre trials)

This reporting is done by using the appropriate SAE form. For the contact details, see below.

It is the responsibility of the local Principal Investigator to report the SAE’s to the local EC(s).

It is the responsibility of the Trial Bureau to report the SUSAR’s to the EC(s) and CA’s according to national legislation

In case the investigator decides the SAE is a SUSAR (Suspected Unexpected Serious Adverse Reaction), the Trial Bureau will report the SUSAR to the Central EC and the CA within the timelines as defined in national legislation. The National Coordinating Investigator reports the SUSAR to all local Principal Investigators.

The first report of a serious adverse event may be made by telephone, e-mail or facsimile (FAX).

Contact details of the Trial Bureau:

e-mail: [Trialbureau@uzgent.be](mailto:Trialbureau@uzgent.be)

tel.: 09/332 89 99

fax: 09/332 89 90

Contact details of the National Coordinating Investigator:

e-mail: [Annemie.dhondt@ugent.be](mailto:Annemie.dhondt@ugent.be)

tel.: 09/332 45 79

fax: 09/332 45 99

The investigator must provide the minimal information: i.e. trial number, subject's initials and date of birth, medication code number, period of intake, nature of the adverse event and investigator's attribution.

This report of a serious adverse event by telephone must always be confirmed by a written, more detailed report. For this purpose the appropriate SAE form will be used. Pregnancies occurring during clinical trials are considered immediately reportable events. They must be reported as soon as possible using the same SAE form. The outcome of the pregnancy must also be reported.

**If the subjects are not under 24-hour supervision of the investigator or his/her staff (out-patients, volunteers), they (or their designee, if appropriate) must be provided with a "trial card" indicating the name of the investigational product, the trial number, the investigator's name and a 24-hour emergency contact number.**

Annual Safety Reporting

The Trial Bureau will ask the National Coordinating Investigator for an annual report containing an overview of all SSARs (Suspected Serious Adverse Reaction) and a summary regarding the safety of the trial subjects. The Trial Bureau will send this report to the Central EC and the CA within the timelines as defined in national legislation. The National Coordinating Investigator will pass this annual report to all local Principal Investigators.

# Studie-analyse

## Berekening steekproefomvang

De beslissing om 20 patiënten te onderzoeken is gebaseerd op beschikbaarheid van patiënten die voldoen aan inclusiecriteria en haalbaarheid.

## Analyse van de stalen

Bij de bepaling **antitrombine** (AT) activiteit in citraatplasma wordt plasma geïncubeerd met een overmaat aan factor Xa (FXa) in aanwezigheid van heparine. FXa vormt een complex met heparine en AT. De niet gebonden FXa komt tussen in de hydrolyse van een chromogeen substraat, nl. S-2765 tot pNA. De pNA vrijstelling wordt gemeten bij 405 nm. Deze vrijstelling is omgekeerd evenredig met de AT activiteit in het plasma.

Kwantitatieve bepaling van **laag moleculair gewicht heparine** (LMWH) in citraatplasma berust op het principe dat LMWH een complex vormt met antitrombine. Daardoor neemt de anti-FXa-activiteit van antitrombine toe. FXa wordt in overmaat toegevoegd aan een mengsel van ongedilueerd plasma en het chromogeen substraat S-2732.

De concentratie van pNA is omgekeerd evenredig aan de LMWH/heparine/andere concentratie in het staal.

**Trombinegeneratietesten** worden uitgevoerd met de Calibrated Automated Thrombinography-methode. Daarbij wordt de totale hoeveelheid trombine die gevormd wordt gemeten. De bepaling gebeurt in plaatjesarm citraatplasma met toevoeging van tissue factor en fosfolipiden.

**CRP** analyse betreft een particle-enhanced imunoturbidimetrische assay.

Anti-CRP antilichamen zijn gebonden op latexpartikels. Deze antilichamen reageren met CRP in het staal met de vorming van antilichaam-antigen complexen. Daardoor agglutineren de latexpartikels met vertroebeling van het staal. Deze troebeling wordt dan turbidimetrisch gemeten.

In EDTA bloed wordt het aantal rode bloedcellen geteld (impedantie). Het **hematocriet** wordt gemeten door de cumulatie van de verkregen pulsen bij telling van de rode bloedcellen.

**Ureum** wordt bepaald met een standaard labo-methode nl. enzymatische reactie gevolgd door een spectrofotometrische analyse.
**Beta2** **microglobuline**: wordt gekwantificeerd met een sandwich ELISA (firma Orgentec Diagnostika GmbH,
Mainz, Germany)

Ureum en beta2 microglobuline worden bepaald in het laboratorium van nefrologie, UZ Gent (prof dr R Vanholder)

Alle andere analyses worden uitgevoerd in het Laboratorium voor Klinische Biologie UZ Gent, op de afdelingen Speciale stolling en Hematologie (Dr. K. Devreese) en Chemie (Dr. J. Delanghe).

## Statistische analyse

Beschrijvende statistiek.

Repeated measures analysis of variance (Friedman). Indien significant gevolgd door Wilcoxon signed rank test. Significantie wordt aanvaard als p<0.05.

De statistische analyse wordt uitgevoeerd door Annemieke Dhondt, Dienst nierziekten, UZ Gent.

# Kwaliteitscontrole- en bewaking

Kwaliteitscontrole van de data in de CRF’s zal gebeuren door vergelijking van de data tussen de brondocumenten en de CRF’s door een persoon die niet betrokken was bij het invullen van de CRF’s.

# Verzekering voor schadeloosstelling

Polis UZ Gent - foutloze aansprakelijkheid

# Publicatiebeleid

Het is de bedoeling de resultaten te publiceren in een A1 tijdschrift. Hierbij zal de privacy van de proefpersonen ten allen tijde gerespecteerd worden.

# Part II : General part of the protocol

# Independent Ethics Committee (IEC) / Institutional Review Bord (IRB)

This trial can only be undertaken after full approval of the protocol and addenda has been obtained from the IEC/IRB. This document must be dated and clearly identify the protocol, amendments (if any), the informed consent form and any applicable recruiting materials and subject compensation programs approved.

During the trial, the following documents will be sent to the IEC/IRB for their review:

- reports of adverse events that are serious, unexpected and associated with the investigational drug
- all protocol amendments and revised informed consent form (if any).

Amendments should not be implemented without prior review and documented approval / favorable opinion form the IEC/IRB except when necessary to eliminate an immediate hazard to trial subjects or when the change involves only logistical or administrative aspects of the trial.

Reports on, and reviews of the trial and its progress will be submitted to the IEC/IRB by the investigator at intervals stipulated in their guidelines.

At the end of the trial, the investigator will notify the IEC/IRB about the trial completion.

# ICH/GCP guidelines

This trial will be conducted in accordance with the protocol, current ICH-GCP guidelines and applicable law(s).

Good Clinical Practice (GCP) is an international ethical and scientific quality standard for designing, conducting, recording and reporting trials that involve the participation of human subjects. Compliance with this standard provides public assurance that the rights, safety and well-being of trial subjects are protected, consistent with the principles that have their origin in the Declaration of Helsinki, and that the clinical trial data are credible.

# Subject information and informed consent

Prior to entry in the trial, the investigator must explain to potential subjects or their legal representatives the trial and the implication of participation. Subjects will be informed that their participation is voluntary and that they may withdraw consent to participate at any time. Participating subjects will be told that their records may be accessed by competent authorities and by authorized persons without violating the confidentiality of the subject, to the extent permitted by the applicable law(s) and/or regulations. By signing the Informed Consent Form (ICF), the subjects or legally acceptable representatives are authorizing such access.

After this explanation and before entry to the trial, written, dated and signed informed consent should be obtained from the subject or legally acceptable representative. The ICF should be provided in a language sufficiently understood by the subject. Subjects must be given the opportunity to ask questions.

The subject or legally acceptable representative will be given sufficient time to read the ICF and to ask additional questions. After this explanation and before entry to the trial, consent should be appropriately recorded by means of either the subject's or his/her legal representative's dated signature or the signature of an independent witness who certifies the subject's consent in writing. After having obtained the consent, a copy of the ICF must be given to the subject.

In case the subject or legally acceptable representative is unable to read, an impartial witness must attest the informed consent.

Subjects who are unable to comprehend the information provided or pediatric subjects can only be enrolled after consent of a legally acceptable representative.

# Case Report Forms

The source documents are to be completed at the time of the subject’s visit. The CRFs are to be completed within reasonable time after the subject’s visit.

The investigator must verify that all data entries in the CRFs are accurate and correct. If certain information is Not Done, Not Available or Not Applicable, the investigator must enter "N.D." or "N.AV." or "N.AP", respectively in the appropriate space.

# Direct access to source data / documents

The investigator will permit trial-related monitoring, audits, IRB/IEC review, and regulatory inspection(s), providing direct access to source data/documents.

# Data handling and record keeping

The investigator and sponsor specific essential documents will be retained for at least 20 years. At that moment, it will be judged whether it is necessary to retain them for a longer period, according to applicable regulatory or other requirement(s).

# Signature page

*Investigator:*

Name: ________________________________________________

Title: ________________________________________________

Signature: ________________________________________________

Date: ________________________________________________

*Investigator:*

Name: ________________________________________________

Title: ________________________________________________

Signature: ________________________________________________

Date: ________________________________________________
